# Mapping the Social Organisation of Neglect in the Case of Fibromyalgia: Using Smith's Sociology for People to Inform a Systems‐Focused Literature Review

**DOI:** 10.1111/1467-9566.70008

**Published:** 2025-01-31

**Authors:** Caroline Cupit, Teresa Finlay, Catherine Pope

**Affiliations:** ^1^ Nuffield Department of Primary Care Health Sciences University of Oxford Radcliffe Observatory Quarter Oxford UK

## Abstract

Fibromyalgia is a syndrome characterised by persistent unexplained pain and fatigue. People with fibromyalgia report receiving little support to manage symptoms, difficult interactions with healthcare practitioners and stigma associated with this contested condition. In this article, we employ Dorothy E Smith's Sociology for People to undertake a systems‐focused literature review from the standpoint of people with fibromyalgia, moving beyond individual subjectivities to map how problems are socially organised. This is a novel application of a Sociology for People which, although previously used to structure research projects, has not previously been reported as a framework for literature review. Our findings highlight how, within a biomedically orientated healthcare system, practitioners' activities are organised to withdraw support from people with fibromyalgia and characterise problems as “psychological”. Those looking to make service improvements for this patient group need to specifically challenge biomedical systems and ideology, in order to promote alternative models of care. We highlight a Sociology for People as a powerful lens for systems‐focused literature review that links frontline experiences with dominant power relations, and provides an alternative to traditional qualitative evidence syntheses. Additionally, the theoretically‐grounded and creative use of published literatures is an ethical approach adding value to extant research.

## 1 Introduction

Fibromyalgia is a catch‐all diagnosis (Jutel 2010, 230) based on a cluster of symptoms, particularly chronic widespread pain and fatigue, sleep disturbance, impaired cognitive and physical function and psychological distress (National Institute for Health and Care Excellence (NICE), 2021; Royal College of Physicians 2022). Diagnosis often takes months or years and involves multiple appointments across different medical specialties to exclude other pathology (Boulton 2019). The condition continues to be marked by considerable uncertainty about aetiology, diagnosis and treatment (Arnold et al. 2016; Mengshoel et al. 2018)—although new theories and treatment models are emerging (Royal College of Physicians 2022).

People diagnosed with fibromyalgia consistently report feeling overlooked and/or having their concerns dismissed by practitioners. They face burdens of treatment (e.g. medication side effects) and non‐treatment (e.g. coping without solutions) (May et al. 2014) and spend much time and effort fighting to get a diagnosis (Dumit 2006), and attending repeated appointments (Madden and Sim 2016; Hintz 2022; Byrne et al. 2023). They have to make sense of their condition (Sallinen and Mengshoel 2019) and cope with disappointment when treatments fail to resolve symptoms. Many report stigma, marginalisation or implicit accusations of laziness (Mengshoel et al. 2018; Doebl, Macfarlane, and Hollick 2020). These burdens are compounded by aetiological and treatment uncertainties (Jutel 2010; Stone 2014). Mengshoel et al. (2018) show that the frequent failure of diagnosis to validate and make sense of the condition results in some patients attempting to negotiate with practitioners, family and social networks about naming and framing diagnosis and treatment (Durif‐Bruckert, Roux, and Rousset 2015; Madden and Sim 2016; Armentor 2017). Some fight against diagnosis, reframing their illness using other more biomedically legitimate labels (Brown 2022). Others struggle to manage diagnostic disclosure controlling “when and to whom [they] talk about [their] illness” (Agyare 2020, 113). Disillusionment with healthcare interactions provokes a “chaotic” search to make sense of the situation (Byrne et al. 2023), and a “trial‐and‐error process to find [other] effective treatment and more appropriate coping strategies” (Mengshoel et al. 2018, 204). This may involve consulting multiple clinicians, and accessing services outside mainstream healthcare services.

Patient accounts, such as those highlighted above, are typically reported in the social science literature, and often foreground individual subjectivities. These are crucial in making visible the difficulties experienced by people with fibromyalgia. However, such experiential foci often leave little room to address questions relating to *why* these problems occur consistently across healthcare—and *how* they may be organised to happen.

In this paper, we use Smith's (2005) *Sociology for People* to interrogate how individual subjectivities are socially organised. We specifically focus on how it comes to be that people with fibromyalgia are frequently given the message that “there's nothing wrong” (Madden and Sim 2016, 99; Agyare 2020, 156), despite presenting with debilitating pain and fatigue, and despite often having a diagnosis that *should* facilitate access to useful therapies (Macfarlane et al. 2017). Our system‐focused review draws on a Sociology for People's analytic tools to “bring to light new ways of looking at a set of primary research studies” (Suri and Clarke 2009, 406) and seeks to broaden the (fibromyalgia) research agenda by examining institutional coordination. Through an iterative exploration of connected literature—moving between patient and professional experiences and sociological critiques (Quirke and Gaudillière 2008)—we investigate how neglect is socially organised for people with fibromyalgia. Although some previous work has drawn attention to the power structures involved in the classification of illness and disease (Dumit 2006; Aronowitz 2008; Armstrong 2011; Jutel 2011), there has been little analysis of the ruling relations governing frontline practices of fibromyalgia diagnosis and treatment (the “extra‐local” or “institutional” spheres of “human action and coordination” (Bisaillon 2012, 618)). Our intention is to map key coordinating systems, focussing attention on the practical, socially organised and material workings of biomedicine, and highlighting opportunities for targeting intervention at a system level.

## 2 Methods: Using Smith's Sociology for People to Conduct a Systems‐Focused Literature Review

Dorothy Smith's Sociology for People (Smith 2005), also described as institutional ethnography (IE), is a well‐established framework for primary research which attends to the power relations that organise what happens in people's everyday experiences. Theory and methods are detailed in a now extensive corpus of work (e.g. Smith 1987, 1990, 2005, 2006; Campbell and Gregor 2002; Griffith and Smith 2014) and have previously been reported in this journal (e.g. Cupit et al. 2020). However, here we apply Smith's conceptual tools to inform an inductive, systems‐focused literature review, starting in the standpoint of people with fibromyalgia. This review was conducted in preparation for work on the “Patient Centred Care for Fibromyalgia: New Pathway Design” (PACFiND) project (University of Aberdeen 2024), explaining our particular interest in UK healthcare.

As we detail below, our use of a Sociology for People to frame our review contrasts with qualitative evidence synthesis approaches such as meta‐ethnography, critical interpretive synthesis and meta‐narrative (Barnett‐Page and Thomas 2009). Qualitative evidence synthesis “brings together the findings from primary qualitative research in a systematic way” (Flemming and Noyes 2021, 1). Here, we have not attempted a systematic synthesis to aggregate findings or develop new theories. Instead, we used Smith's Sociology for People to first extract evidence of people's “doings” from a body of literature, and then investigate how specific problems are socially organised, from wider reading and analysis (Smith 2021, 76). We employed Smith's ontological framework, drawing on *published research literature* to gain insight into the lives and healthwork of people with fibromyalgia—an often invisible field of activity (Webster et al. 2023). Crucially, we then also followed “trails” (Griffith and Smith 2014, 8) into the institutional domain, producing a systems‐focused “tale of the field” (Van Maanen 1988, 1) that draws on qualitative research literature. Consequently, the product of our analysis serves a different purpose from other reviews which might, at first sight, seem to cover similar ground (e.g. Mengshoel et al. (2018) meta‐ethnography on experiences of fibromyalgia diagnosis).

As it is central to a Sociology for People, our review starts with a commitment to taking a standpoint (Campbell and Gregor 2002, 48), and explicating social relations from that standpoint. The researcher is committed to “taking sides”—producing an account that is primarily a resource for the standpoint group (Smith 1987, 177; Cupit, Rankin, and Armstrong 2021)—whilst also showing how ruling relations regulate the activities of others. For this reason, we start our review with research literature that specifically *foregrounds the difficulties faced by people with fibromyalgia as they come into contact with healthcare services*. We use this analysis to develop an illustrative map as the basis for a critique of institutional relations.

### 2.1 Practical Approach to Systems‐Focused Literature Review

In practical terms, we followed processes described by Smith and colleagues, using an iterative process of (1) collating and attending to the work of standpoint informants, focussing on what people with fibromyalgia have to do as part of their interactions with healthcare, and what happens in frontline clinical practice; (2) developing guiding problematic (3) explicating problems by drawing on practitioner accounts; (4) aligning relevant critical analyses; (5) producing an illustrative map showing key ruling relations; and (6) corroborating findings with people diagnosed with fibromyalgia. These steps follow well‐established ways of using Smith's ontology in primary research. Although we do not aim for replicability, we describe how we proceeded, as our process may be useful to other researchers. Bisaillon's (2012) analytic glossary of terms may be helpful for readers unfamiliar with Smith's Sociology for People.

#### 2.1.1 Collating and Attending to Standpoint Informants' Work and Its Organisation

We conducted database searches in April 2021, and updated these on two other occasions during our analysis. We used the Scopus database to run initial searches for relevant literature over the previous 10 years, supplementing this with Google Scholar searches and forward/backward reference chaining. We used various search terms (e.g. “patient” “experience” “fibromyalgia” “health services”) and scanned abstract/full text to identify publications which foregrounded the accounts of people with fibromyalgia, and their experience of healthcare in the UK. Our searches identified one meta‐ethnographic review of the diagnostic experience of patients with fibromyalgia (Mengshoel et al. 2018), and a core set of 10 additional papers (Agyare 2020; Brown 2022; Diver, Avis, and Gupta 2013; Van Gordon, Shonin, and Griffiths 2016; McMahon et al. 2012; Madden and Sim 2016; Boulton 2019; Lempp et al. 2009; Doebl, Macfarlane, and Hollick 2020; Dennis, Larkin, and Derbyshire 2013), which forms the basis of our review. One of these papers included people with fibromyalgia from Canada as well as the UK (Boulton 2019).

Our analysis involved multiple readings of the selected papers, looking for traces of people's “work”, “disjunctures” and “social organisation” using Smith's articulation of these concepts. In the first instance, our focus was particularly on the work undertaken by people with fibromyalgia, using Smith (2005, 229) “generous concept” that defines work as “anything that people do that takes time, effort and intent”. Smith advocates this focus on work as an alternative to the creation of *themes* (J. Rankin 2017). We looked for evidence in patients' accounts (in the literature) about what they were doing, how their activities intersected with practitioners' work and how texts were mediating, regulating and authorising people's activities (Smith 2001; Cupit, Rankin, and Armstrong 2021). Our reading of empirical studies focused on material work that was “easily envisioned” (Best and Marcus 2009, 10) and we did not seek to reinterpret findings or undertake reciprocal translation (as in meta‐ethnography).

To illustrate our attention to *work* we take an example from Diver, Avis and Gupta (2013) who present four truncated vignettes selected to represent particular illness narratives (Frank 1997). As our aim was to focus on people's tangible *work* rather than on their individual sense‐making in relation to illness, we highlighted data from the narrative accounts that pointed specifically to that work, both in direct quotations and the authors' commentary. Thus, within one individual's narrative, there were references to a variety of physical and mental activities, such as making adaptions to her schedule to reduce tiredness, attending multiple medical appointments and trying to legitimise her symptoms when tests results were normal. Her narrative showed that considerable time and effort was spent pursuing a diagnosis (which was not actively pursued by medical practitioners) in order to gain credibility and understanding. We coded this as *legitimacy work*. By conceptualising such efforts as *work*, we were able to ask why it was so important and so stressful for people—a thread that ran throughout our selected literature. What was going on for these people? How were their efforts being organised? Although authors such as Driver et al. suggest explanations relating to, for example, individual *identity* issues, their goal was not to probe questions about work per se. However, using Smith's lens we were prompted to consider how such work might be hooked into systems of social organisation. As we highlight below, using Smith's Sociology for People as our review framework led us to consider how legitimacy work (for example) was orientated to the work of others, such as health practitioners.

We also looked for traces of guiding *texts* (“material artefacts that carry standardising messages” (Bisaillon 2012, 620)) and *discourse* (“a systematic way of knowing something that is grounded in expert knowledge and that circulates widely in society through language, including most importantly language vested in texts” (Mykhalovskiy 2002, 39)). As Smith and others have highlighted, these textual systems of coordination are often ignored in more conventional anthropologically‐influenced research. This is because their production and circulation is choreographed by policymakers and administrators whose work is remote from local service delivery (Smith and Turner 2014). Within this managerial domain, people develop electronic codes, templates, economic models, policy documents, or artificial intelligence algorithms (for example)—activity that may be invisible to both frontline staff and recipients of care, and which can appear uninteresting, too complex or out‐of‐scope to a fieldworker. Although these textual processes are often unexamined in patient‐experience research, they are frequently mentioned and can be recognised and explored.

#### 2.1.2 Developing a Guiding Problematic

Having identified various patterns of work in our selected papers, we consolidated our analytic interest into a *problematic* to guide our review. In Smith's ontology, a problematic “directs attention to a possible set of questions that have yet to be posed or of puzzles that are not yet formulated as such, but are ‘latent’ in the actualities of our experienced worlds” (Smith 1987, 89). Our problematic centred on the tension between patients’ often‐desperate search for support (with debilitating symptoms of pain and fatigue) and healthcare systems that seemed unwilling/unable to provide it. Various forms of work that we identified while reading the papers (e.g. legitimacy work) appeared to be directly related to this search for support. We studied in more detail what people did as part of their search for support, and how their needs were or were not addressed by health services.

#### 2.1.3 Explicating Problems by Drawing on Practitioner Accounts

In order to explicate our problematic (to sketch the dominant ruling relations that organise people's troublesome search for support), we looked for what practitioners did when people presented with symptoms that could not be easily explained. As this was not the primary focus of any of the originally‐selected studies, we undertook additional searches to identify practitioner accounts of working with people with fibromyalgia (using Scopus and Google Scholar). It became increasingly clear that the difficulties practitioners reported were (from their perspective) related to the unexplained nature of the symptoms, so we then expanded our search to include not only fibromyalgia but also a wider range of unexplained symptoms. From reading these practitioner‐focused papers, we made connections between patients' difficulties in accessing support and practitioners' difficulties with supporting patients in a biomedical system that is increasingly geared to quick and efficient testing and treatment.

#### 2.1.4 Aligning Relevant Critical Analyses

Smith's Sociology for People provided a lens for interpreting the research literature. To support the presentation of our analysis, we sought system‐level critiques which were congruent with our emerging findings. Drawing on our own sociological knowledge, we positioned our analysis within familiar broad critiques of *biomedicine* (see, e.g., Davis and Gonzalez 2016). In doing so, we did not take up an ideological frame (see Smith 2005, 155 on avoiding “ideological capture”). Instead, as Smith frequently has done in her work, we harnessed ideas and language that would support the interests of people with fibromyalgia and allow us to *talk back* to the ruling relations which organise their experience of health services. As Smith stresses in her book, *The Conceptual Practices of Power* (Smith 1990), language is central to both ruling power relations and to activist attempts to resist them.

#### 2.1.5 Producing an Illustrative Map Showing Key Ruling Relations

We developed an illustrative map, sketching the patterns of work identified in our literature review, using the approach developed by Smith (Smith 1990; Griffith and Smith 2014) and adapted in primary research (see Cupit, Rankin, and Armstrong 2021). This involved considering the key texts—representations embedded in language (e.g. viewing people's problems as “psychological”)—to which the major patterns of work appeared to be orientated. The resulting illustrative map is based on Smith's “small hero” diagram (Smith 2006, 3), which attempts to convey the relationship between an individual's standpoint location and governing systems of social organisation. It is important to note that this map was not produced to be an authoritative or single way of knowing about the social organisation of problems faced by people with fibromyalgia, but to provide an analysis orientated to the concerns and interests of people with fibromyalgia. Our intention was to move beyond subjectivities to illuminate material and discursive forms of social organisation.

**FIGURE 1:**
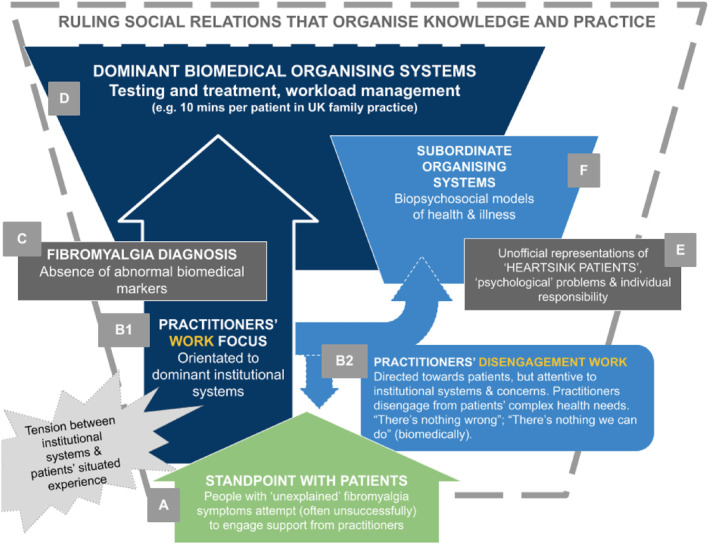
The social organisation of neglect following fibromyalgia diagnosis: a systems‐focused map.

#### 2.1.6 Reflexivity and Corroboration of Our Review Findings With Patients

Applying Smith's Sociology for People requires considerable conceptual discipline. For example, our standpoint with people diagnosed with fibromyalgia forces an explicit positionality, which guided the whole review, including our focus on materially‐organised systems. In line with Smith's approach, our analysis during steps 1–5 explicated problems reported consistently by patients—interrogating how and why these problems so routinely occurred and how circulating discourse and texts shaped practice.

By carefully adhering to Smith's guiding ontology and conceptual tools, we were to some extent insulated from being drawn into our own researcher‐focused interests and subjectivities. Nevertheless, our exploration was shaped by our existing knowledge and experience of healthcare systems, and our expertise in sociological analysis. The findings and interpretations offered in our review are open to test, challenge and refinement—as applies to all research outputs. However, we are confident that this review provides a useful resource for people with fibromyalgia and their advocates, providing insight into organising forces that shape what happens in local practice and that may be amenable to intervention and/or the subject of further research.

Specifically, the findings of our review are supported by people with fibromyalgia (and also by health professionals reviewing this manuscript). Throughout the review process, we listened to Patient and Public Involvement and Engagement representatives on the PACFiND steering committee and also paid attention to contemporaneous experiences of people with fibromyalgia (with their permission), through social media support groups. These contemporaneous connections with fibromyalgia patients enabled us to cross check that the concerns represented in the literature were congruent with the concerns of the communities with which we were engaged, and to sense‐check our review findings as we developed them. Additionally, CC published a conference presentation online, and was contacted by three people with lived experience of the condition, who strongly supported the review findings as offering explanations for their own experiences.

### 2.2 Methodological Clarifications

Before proceeding to our findings, we address two issues that relate to our approach and our terminology.

First, there is a widespread (and, in our view, unhelpful) dogma among some of Smith's followers that literature review is universally dangerous, owing to the institutional (ideological) knowledge that it produces and perpetuates. This seems to stem from Smith's work to expose academia (including the way researchers produce and enshrine particular theoretical framings) as a key sphere of ruling activity. Specifically, her most prominent critique was aimed at core sociological texts written by men (e.g. Giddens), which she came to understand as regulators of women's experience (Smith and Turner 2014). Starting from her own experience (and taking an academic woman's standpoint), she highlighted the power of such texts to “insulate” against women's subversive voices, through ideas and forms of language that excluded their experiences (Smith and Turner 2014, 248). Smith's critique of sociology in the context of the women's movement in 1970s/80s has led many of her followers to apply an almost universal and unnuanced scepticism to all academic research, willing only to analyse the concepts, forms of knowledge and theoretical discourse that it conveys. Although sharing Smith's attention to academia as *active discourse* (Mykhalovskiy 2001), and recognising the oppressive potential of research to subdue the interests of particular groups (in this case, people with fibromyalgia), we contend that there is significant value to social science literature that gives voice to underrepresented groups (including, but not only, research undertaken using Smith's approach). The literature we use is, of course, mediated through the analytic lens of the researchers/writers, and shaped by academic processes for selecting and presenting data. However, with Smith, we contend that when handled with care, people's experiences as presented in research literature can be a valuable resource to highlight common everyday problems and, from that starting point, can support a productive analysis of ruling relations. Moreover, such an approach also honours the contribution of previous study participants by attending to their voices. Smith herself drew on a wide range of academic and other publications to develop her Sociology for People, and support her standpoint‐orientated investigations (Stanley 2018). Her extensive academic writing, lectures and informal discussions clearly show that she was *selective* in how she highlighted problematic framings in the literature, and that she was opposed to universal prohibitions and/or a dogmatic application of her ideas.

Second, we are careful in our use of the terms “ethnography” and “IE”. We have chosen to use Smith's term “A Sociology for People” rather than the analogous “IE” to emphasise that we are employing her *complete framework* for conceptualising power relations and how people engage with them. This is important because Smith's approach uses the term “ethnography” differently from many social scientists. As “ethnography” is often misused as a synonym for participant observation, we must be clear that our review has not involved fieldwork in physical spaces. Instead, we have employed Smith's ontology to frame our exploration of the social organisation of difficulties faced by people with fibromyalgia as presented in the research literature. Our iterative approach to literature selection, analysis, and reporting reflects Smith's approach to focused “inquiry” (Smith 2005, 3). This includes elements of more anthropological ethnographic practice, for example, prioritising an emic perspective (an insider's view of managing fibromyalgia symptoms), immersing ourselves (in the literature) and investigating how various power structures (including processes for collecting and using evidence) pattern local practices (Savage 2006).

## 3 Findings: Biomedicine and the Production of Neglect

In this section, we illustrate how biomedical systems of healthcare coordinate the experiences of people with fibromyalgia. *Biomedicine* is used to describe the dominant approach to healthcare in which physiology is the basis for understanding mechanisms of illness and developing strategies for prevention, diagnosis and treatment. Biomedicine is “a framework, a set of philosophical commitments, a global institution woven into Western culture and its power dynamics” (Valles 2020, 1) which privileges single disease mechanisms and fixing people with (mostly pharmaceutical) interventions (Davis and Gonzalez 2016). We draw selectively and iteratively on connected literatures, using the term “biomedicine” as descriptor for dominant and overarching ruling relations. As highlighted in the introduction, we are focused on the common experience of people with fibromyalgia, who report that practitioners have told them that there is “nothing wrong” and/or that “there's nothing we can do”. We draw on the philosopher, Annemarie Mol (2008, 97), writing on care—and her contrast with “neglect” or “abandonment”.

We present our findings in four sub‐sections. First, we investigate patient accounts of what happens in interactions with practitioners, and highlight a disjuncture between patients' experiences and the biomedical systems with which they collide. Second, we make connections with corresponding accounts of practitioners, who work to define who can and cannot be helped within increasingly efficiency‐driven (biomedical) systems. Third, we highlight the additional work, that is, generated for practitioners and patients as a consequence of biomedically orientated systems—specifically the need for practitioners to withdraw from supporting patients with fibromyalgia. This draws on additional critiques of Engel's (1981) biopsychosocial model and patient empowerment discourse to highlight the biopsychosocial model not as an *alternative* to biomedicine but as *subservient* to it. Fourth, we illustrate how biomedical systems practically coordinate practitioners' knowledge and work, and actively produce the conditions by which the needs of people with fibromyalgia are neglected.

### 3.1 From a Patient Standpoint: Unexplained Symptoms in a Biomedical System

Patients consistently report that they have been told by practitioners that there is “nothing wrong” with them (e.g. Madden and Sim 2016). As Boulton (2019) argues, based on interviews with people with fibromyalgia, this is a diagnosis of “nothing and everything”. It is *nothing* because it is “largely an empty promise [which] fails to provide definitive answers or confer meaning and legitimacy to [patients'] illness experiences” and yet it is also *everything,* a catch‐all diagnosis within which a “multitude of [unexplained] symptoms” can be incorporated. A participant in Boulton's study describes how this “diagnosis of exclusion” is operationalised in everyday clinical practice:

I told the doctor my symptoms and he just immediately said it sounds like fibromyalgia, but it could also be these other things, so we’ll test for everything else and if you don’t have anything we can find then it’s fibromyalgia. I had quite a few tests, like blood tests and scans and things like that, and there was nothing. They couldn’t find anything wrong. So, then he said, “Right well you’ve got fibromyalgia”.

(Participant, Boulton 2019, 812)

In this example, the finding that “there was nothing” arises when biomedical testing is unable to identify a cause for the patient's symptoms. To the patient with pain and fatigue impinging on their ability to work, participate in ordinary family life and so on, this is clearly not “nothing”. This exposes a disjuncture between two fundamentally different ways of knowing—between the systems that coordinate healthcare, and the everyday realities of a patient's experience.

We do not know how the practitioner in this particular example interprets the patient's symptoms, or how biomedical testing influences their perspective. However, we can see that unexplained symptoms do not sit easily within a biomedical healthcare system, founded as it is on the identification of biological anomalies through testing, followed by protocol‐driven (mostly pharmaceutical) care modalities (Davis and Gonzalez 2016).

Agyare's (2020) study of patients' experiences of fibromyalgia provides further illustrations of how the absence of biomedically visible abnormality conflicts with patients' everyday experience of symptoms. One participant notes how frustrating this is:

[…] because I had always been told nothing is wrong but my body wasn’t behaving normally, like they say you are fine but my body wasn’t acting fine.

(Participant 4, Agyare 2020, 103)

In this example, the patient is left to make sense of this contradiction.

The absence of biomedical evidence creates many difficulties for the individual—which are compounded when the fibromyalgia diagnosis prompts a practitioner to halt further investigations and treatment. In one of many examples in Agyare's study, a participant reports that following the fibromyalgia diagnosis, they were told to “go away” to research self‐management:

[The doctor] said “what you need to do is go away and do as much research on it as you can”. He said “there is no cure, here’s a tablet”. He said “you need to learn how to manage it”. And so yeah everything that I have learnt about it, I’ve learnt from the internet.

(Participant 3, Agyare 2020, 109)

The practitioner's comment here that “there is no cure” again alerts us to the biomedical solution focused systems of healthcare (Davis and Gonzalez 2016; Mol 2008). Support is offered in form of “a tablet”, a pharmacological intervention to ease symptoms, which will not address underlying causes. Patients are unsurprisingly dissatisfied by such solutions when these are the only form of treatment—and may contest the approach:

Although you can’t help me with medication there are other ways you can help me

(Participant 1, Agyare 2020, 104)

The literature on patient experiences of fibromyalgia is full of narratives which highlight interactions through which they are “dismissed” following fibromyalgia diagnosis. Doebel et al. (2020) literature synthesis, entitled “No one wants to look after the fibro patient”, highlights patient accounts of inconsistent and poorly coordinated care. This, we argue, is a direct product of systems of biomedicine which have few answers to the unexplained symptoms of fibromyalgia.

As outlined previously, exploring disjunctures directs our gaze beyond the local sites of clinical consultation to biomedical systems of organisation (Clarke et al. 2003; Davis and Gonzalez 2016; Valles 2020; Kroll 2021). These organised systems of management, policy and research coordinate what happens in frontline clinical consultations, but are often invisible to patients and practitioners (Griffith and Smith 2014). In Smith's ontology, biomedical discourse, as a sphere of textually‐organised activity, produces “institutional” or “ideological” knowledge—“ideas and social forms of consciousness [which] originate outside experience […] a forced set of categories into which [people] must stuff the awkward and resistant actualities of [their] worlds” (Smith 1987, 55). This means that, as shown in the excerpts above, practitioners operating within the biomedical frame can find that there is “nothing wrong” whilst simultaneously hearing patients report pain, fatigue, fibro‐fog and a myriad of other symptoms. Biomedical knowledge and practice produce “contextual stripping” of patients' experiences, in which their contextualised everyday stories are overruled (Smith 1990, 137).

### 3.2 Practitioners' Work to Define Who can and Cannot Be Helped

In this section, we consider how practitioners respond to patients with unexplained symptoms. We show how practitioners' and patients' work intersects, and how their work is shaped by strong institutional forces. We highlight some of the systems and processes through which practitioners delineate whose needs to prioritise.

Practitioner accounts suggest that biomedically organised systems create problems for them too. Practitioners deal with “feelings of frustration, helplessness and failure” when faced with patients with unexplained symptoms (Åsbring and Närvänen 2003, 715). Åsbring and Närvänen's study of chronic fatigue syndrome and fibromyalgia highlights that patients can quickly become the focus of that frustration:

It was described as especially troublesome when the patients consulted the same physician several times, despite the fact that, in the physician’s opinion, there was little more that could be done for them.

(Åsbring and Närvänen 2003, 715)

Similarly, Johansen and Risor (2017) review of GPs' experiences of medically unexplained symptoms highlights tensions created when biomedical systems of diagnosis and treatment bump up against the realities of complex illness. They describe how practitioners manage this tension by blaming patients and their *social problems*—over which they perceive themselves to have no influence (Johansen and Risor 2017). Patients are objectified as *heartsink* patients:

Patients were described as “frustrating” or “heartsink”. Exploration of such feelings revealed a spectrum of emotions from inadequacy to resentment and fear of such patients who could dominate and manipulate the course of the consultation.

(Wileman, May, and Chew‐Graham (2002))

This term is controversial but commonplace (O'Dowd 1988; Glass 2019; Salisbury 2022). It locates the problem with patients and reinforces the idea that they cannot be helped. Both the diagnosis of fibromyalgia and this labelling of the heartsink patient form part of biomedicine's textually mediated systems of social organisation (Smith and Turner 2014). The threat posed by heartsink patients is not only to practitioners' self‐esteem, but to the efficient organisation of the wider healthcare system. Biomedically organised, efficiency‐driven systems, compel practitioners to move swiftly from a diagnosis of *nothing* (Boulton 2019) to *doing nothing*, for example, withdrawing support—“there's nothing [we] can do about it” (Diver, Avis, and Gupta 2013, 32). However, disengaging from patients with painful and life‐limiting symptoms may present challenges to practitioners as we will show in the next section.

### 3.3 Facilitating and Legitimising Neglect

Disengaging from patients' health concerns creates a moral challenge (described elsewhere as *moral injury* (Griffin et al. 2019)) for practitioners who understand their role to involve helping people. In this section, we show how practitioners legitimise and reinforce ruling biomedical relations by drawing on ideas from the biopsychosocial model (Engel 1981)—particularly the field of psychology.

Concepts from psychology consistently appear in the social science literature relating to fibromyalgia diagnosis and treatment. Patients report that practitioners tell them that “nothing is wrong”, but simultaneously suggest that their symptoms are “psychological” or “psychosomatic”:

A lot of the doctors, and I have experienced that from the way they’ve spoke to me believe that it is just psychosomatic and that’s it. Didn’t have this trust that you are actually in pain because you can’t prove them with a blood test. There’s nothing tangible to show them that there is something wrong with you, so you must be making it up. Or there must be something wrong with your brain and mental health rather than the actually physical body.

(Participant 5, Agyare 2020, 101)

When practitioners suggest a psychological cause of illness, alongside biomedical discussions of blood tests and so on, they reinforce a Cartesian split between mental and physical health that is frequently used to “trivialise” patients' illness and classify them as “malingerers” or “hypochondriacs” (Mengshoel et al. 2018, 204–205). These judgements also have gendered overtones, as women make up the majority of fibromyalgia diagnoses (ibid.).

Dennis, Larkin, and Derbyshire (2013) highlight the difficulties generated by psychological framings that are severed both from the biological manifestations of illness, and from patients' own accounts:

Psychological accounts carry the threat of additional stigma (via association with “mental illness” discourses). When psychological accounts are presented as if they were dichotomous to physical accounts, they also give the appearance of challenging the legitimacy of the symptoms themselves.

(Dennis, Larkin, and Derbyshire 2013, 779)

Segregating the psychological in this way reinforces a mind‐body dichotomy and obscures the complex body‐mind connections that patients experience:

It’s like a vicious circle, […] your emotion feeds your body, and your body feeds your emotion.

(Participant 1, Agyare 2020, 117)

My body doesn’t let stress out any other way, other than getting sick. I have a tendency to push myself way more than I should, and my body tries to get back at me, I think.

(Participant in Dennis, Larkin, and Derbyshire 2013, 770)

As Lempp et al. (2009, 9) emphasise, “patients clearly pointed out the relationships between body and mind and how pain, insomnia, and specific life events were often related to their mental health”— described elsewhere as the *body–mind* (Berrios 2018) or *mind–body* (Maté and Maté 2022).

Using a Sociology for People, we understand the term *psychological* to function as a “shell term” (Smith 2005, 112) in practitioners' explanations —standing in for people's apparently inappropriate help‐seeking behaviour. The framing of illness as psychological overrides more situated knowledge, drawing attention away from the complex interplay of biological, psychological and social components and positioning patients' help‐seeking as illegitimate. It buttresses the framing of heartsink patients who cannot be supported by established healthcare systems and is infused with morally laden meanings (e.g. people with fibromyalgia lack self‐discipline, cannot cope, are susceptible to somatisation, or cannot control their help‐seeking behaviour). The use of this version of psychology (severed from the bio‐social) adds a new ruling relation into interactions with patients (J. Rankin and Campbell 2006).

The biopsychosocial model of illness (Engel 1981), as popularised in recent years, appears to recognise both the psychological and social dimensions of health and illness but has been critiqued from its inception. Armstrong (1987, 1213, 1217) asserted, for example, that the model was “grossly medicocentric and sociologically naïve”, in effect positioning social science as “an emasculated, uncritical appendage of a reinvigorated biomedicine”. Salmon and Hall (2003, 1973) have since shown how the “psycho” and “social” categories have been employed to cement the “traditional dualism of mind and body through the concurrent construction of patients as “agents in managing their disease” (drawing e.g. on notions of *patient empowerment*). By emphasising a causal relationship between the individual's behaviour and their disease, the biopsychosocial model has often been employed to “locate responsibility for problematic areas of patients' suffering *with the patients*” (ibid., emphasis added).

Thus, we see that the framing of fibromyalgia as psychological performs a vital function within the social organisation of fibromyalgia care. It transfers responsibility from the healthcare system to patients and, in doing so, creates new work for people with fibromyalgia. In particular, this framing infiltrates people's own understandings of their illness identity, prompting them to question their own experiences of illness—a condition described by Smith (1987) as “bifurcated consciousness”. It generates the need for patients to prove not only that their symptoms are real (Lempp et al. 2009) but that they themselves are morally worthy of help (i.e. not time‐wasting (Madden and Sim 2016) or mentally ill (Smith 1978)). Undermining patients' embodied knowledge (of complex mind‐body connections) creates emotional labour (Galvez‐Sánchez, Duschek, and Reyes del Paso 2019) on top of extensive other work to manage illness. It is therefore unsurprising that some people resist psychological understandings and pursue more biomedical explanations (Wainwright et al. 2006; Doebl, Macfarlane, and Hollick 2020)—which fit more easily within dominant ruling relations.

The psychological framing of fibromyalgia can also have significant *material* consequences for patients, extending beyond the immediate health consequences (Aronowitz 2008). One example is in relation to the welfare benefits system. Although a diagnosis of fibromyalgia *may* support a claim to welfare benefits (e.g. Lempp et al. 2009; Diver, Avis, and Gupta 2013; Madden and Sim 2016), recent evidence suggests that welfare assessors may be sceptical about the diagnosis, in part because of this psychological framing:

[…] people who had to give up work, I know they are financially struggling to apply for Universal Credit. Because [government agencies] don’t take this condition seriously enough. So they are actually, they are being penalised for being ill and they don’t have any financial help.

(Participant 7, Agyare 2020, 138)

Consequently, as people with fibromyalgia consistently complain across social media forums, they experience extensive difficulties completing benefits assessments, agonising over how to complete the forms to secure welfare benefits. This work forms a major distraction from the extensive efforts that are already needed to improve their health.

### 3.4 Mapping the Social Organisation of Neglect

We now illustrate the social organisation of neglect following fibromyalgia diagnosis (Figure 1), using the approach outlined by Cupit, Rankin, and Armstrong (2021).

The figure should be read from bottom to top. It starts with the real‐world problematic that guided our analysis—the tension produced when patients attempt to engage support from health practitioners [A], but are told that there is “nothing wrong” [B]—despite obvious indications to the contrary. We have argued that, taken as a whole, practitioners' work focus [D] is orientated to reductionist biomedical systems of diagnosis and treatment—and patients with fibromyalgia do not easily fit into this fixing schema (Davis and Gonzalez 2016). When practitioners are faced with repeated visits from patients with unexplained symptoms, their response is shaped by ruling systems organised around diagnostic testing, and short, single‐condition, test‐and‐treat‐focused appointments (McCartney 2014) [C], [E]. The experience, for practitioners as well as patients, is troubling as tensions between institutional and situated knowledge are exposed. Having to somehow manage the patient in front of them, practitioners are drawn into new forms of work—described here as *disengagement work* [B]. Disengagement work involves prioritising knowledge from biomarker testing [C] to understand the situation (“there's nothing wrong”). To legitimise disengagement, practitioners then activate concepts from psychology and neoliberal economics to assemble an institutional “story” (Smith 1990, 137), in which people with fibromyalgia are represented as “heartsink patients” [F] whose needs are beyond the remit of health services (“there's nothing we can do”). Such framings are supported with reference to the biopsychosocial model (Engel 1981) [G] which, in practice, is positioned as *subordinate* to governing systems of biomedicine [E] rather than as an *alternative* approach.

## 4 Discussion

Smith's Sociology for People provides a novel and valuable approach for systems‐focused literature review. Using this frame, we have highlighted that biomedicine is not only a set of philosophical commitments (J. Davis 2016; Valles 2020) but is also the managerial material systems that shape what happens in interactions between health practitioners and patients. Our goal has been to shed light on these latent systems of accountability (Griffith and Smith 2014, 15), highlighting the tangible connections between problematic patient experiences and large scale institutional infrastructure. We have used the term “biomedicine” (Quirke and Gaudillière 2008) to characterise those ruling relations that socially organise the withdrawal of support from people with difficult‐to‐treat fibromyalgia symptoms. In doing so, we refer to a rich history of social critique in relation to medical knowledge and power (Wright and Treacher 1982), and understand biomedicine to include bureaucratic systems (e.g. for organisation of clinic appointments), as well as systems that directly relate to clinical decision‐making. Using Smith's Sociology for People, we have explicated biomedicine's operation as an *organised sphere of knowledge and activity*, and mapped how those ruling relations configure local practice. Our illustrative map may be useful to those seeking to improve services, by directing attention to the governing systems of biomedicine which influence local service delivery, and highlighting the need for research on *how systems work*, as well as *how people experience* illness and healthcare.

Ruling systems standardise practitioners' work across time and space and ensure that certain forms of practice are prioritised over others. Their generalising and pervasive operation can be seen in the consistent patterns of neglect that we identified in the research literature we reviewed. However, social systems are not totalising. Not *everyone* with fibromyalgia is “neglected”, and practitioners also frequently experience highly problematic tensions in relation to the systems within which they work. Even if they were so‐inclined, practitioners *are not allowed* to completely withdraw support from patients with problems that are considered psychological—and their practices will inevitably vary according to, for example, their professional role or experience. Drawing on Bakhtin (1986), Smith (1996) highlights that, at a local level, people (e.g. practitioners and patients) enter into dialogue with institutional systems and processes—and can exercise agency in relation to them. Thus, individuals and groups may subvert, resist or innovate (Cupit 2022) to differing degrees. Nonetheless, providing support for people with fibromyalgia can be extremely challenging within the existing governing infrastructure—however committed to good care a practitioner may be.

Our analysis suggests that the solutions to managing fibromyalgia that are often proffered within the social science literature (e.g. Sociology of Diagnosis) will prove difficult to action without deliberately and explicitly challenging systems of biomedicine. For example, Boulton (2019, 817) argues that “it might be beneficial, for both patients and doctors, to understand medical encounters as “help‐seeking” interactions, and to recognise that diagnosis is not the ‘be‐all and end‐all of the medical consultation’“. She continues that “this might allow for more opportunities—beyond the diagnosis—for patients and medical professionals to work together to find liveable ways to manage chronic illness.” Similarly, Doebl et al. (2020, 1722) emphasise that “patients [prefer] open and patient‐centred communication styles by health care professionals that [allow] reciprocal information sharing, increased mutual understanding, and [encourage] shared decision‐making about care”. Hauser and Fitzcharles (2018, 58) highlight the need for more connected multi‐disciplinary support for people with complex conditions such as fibromyalgia, arguing that “whether fibromyalgia is a helpful or unhelpful diagnosis […] depends on the information given to the patient regarding the nature of the disorder, planned treatment strategy, and expected outcome after the initial diagnosis”. They highlight the importance of the mind‐body integration in such explanations, emphasising that patients should be informed of the impact of biological, psychological and social factors on the “predisposition, triggering, and perpetuating of fibromyalgia symptoms”. These findings resonate with developments in neuroscience popularised by writers such as Gabor Maté (Maté and Maté 2022) and with recommendations more than a decade ago that “taking a less medical but more holistic approach when drawing up new diagnostic criteria for fibromyalgia might better match individuals' somatic and psycho‐social symptom profile and may result in more effective treatment” (Lempp et al. 2009, 10). Genuinely holistic approaches have been described as the essence of “good care” (Mol 2008) and are particularly important when there are no quick‐fix solutions within the established biomedical repertoire (Davis and Gonzalez 2016). However, despite policy rhetoric advocating individualised care (see, e.g., NICE 2021), interconnected support does not easily fit within siloed institutional structures that manage mind and body separately (Thiabut 2018).

We have illuminated a small part of the institutional complex involved in organising care for people with fibromyalgia in the UK. It is not the entire picture; other analyses that emphasise different and intersecting accountability systems would add to, and perhaps refine, the ideas presented here. We anticipate that the findings of our review, although focused here on fibromyalgia care in the UK, may be applicable to other developed healthcare systems and/or other similar conditions, where similar organising forces are in play. Further work might focus investigation within (for example) the institutional domains of policymaking and commissioning of services, highlighting service structures and workforce (Dennis, Larkin, and Derbyshire 2013), the (in)accessibility of particular services (Briones‐Vozmediano et al. 2013; Doebl, Macfarlane, and Hollick 2020) and intersections with the welfare benefits system (Madden and Sim 2016). It might investigate how institutional systems impact differently on people experiencing particular forms of disadvantage. Further work might also study the work of innovators who are managing to provide new models of care for people with fibromyalgia—working around or resisting the dominant systems of knowledge that govern their practice (Talbot 2020; Cupit 2022). Other directions include research into how practitioners are incorporating developments in neuro‐physiology into their practice and explanatory mechanisms that connect (or disconnect) mind and body (Gatchel and Neblett 2018; Thibaut 2018; Maung 2019).

Our review provides a demonstration of how Smith's Sociology for People can be applied as novel approach to literature review. We have been able to examine the material difficulties that people with fibromyalgia face, and then move onto a systems‐focused analysis of how these difficulties arise. Connecting local practices with large‐scale organising systems is rarely attempted in literature review, but we have shown that this can be productively undertaken to shine a light on significant health service challenges. A strength of this approach is that it has allowed us to iteratively review the literature rather than follow the more rigid protocolised approaches that are favoured in systematic reviews and are typically promoted by those who wish to replicate processes for rigour in biomedicine. The dominance of such protocolised approaches constrains how existing literature can be read, discussed, and harnessed for change. We argue that new frameworks for literature review, such as a Sociology for People, have much to offer the sociology of health and illness.

## 5 Conclusion

Our review has highlighted that the neglect of patients with unexplained fibromyalgia symptoms is socially organised through institutional systems described as biomedicine. Practitioners are bound to orientate to these systems, and employ ideological framings from psychology to legitimise their withdrawal of support. Patients are left with the task of finding relief from debilitating everyday symptoms that, in turn, generate new difficulties on top of their original symptoms. Smith's Sociology for People has enabled a systems‐focused literature review that connects patient experiences and overarching institutional systems. Our analysis is congruent with contemporary experiences of patients and practitioners, and provides an alternative view to those produced previously in the social science literature. We argue that Smith's analytic frame offers a powerful resource for those undertaking research with an activist aim, enabling researchers to map institutional systems and processes that may require further investigation or intervention. We anticipate that our findings may have application to the management of other unexplained symptoms or contested conditions. The approach also has significant potential to add value to extant research literatures.

## Author Contributions

**Caroline Cupit:** conceptualization (lead), methodology (lead), writing–original draft (lead), formal analysis (lead), writing–review and editing (lead). **Teresa Finlay:** funding acquisition (supporting), analysis (supporting), review and editing (supporting). **Catherine Pope:** funding acquisition (lead), conceptualization (supporting), writing–review and editing (supporting).

## Ethics Statement

This study was based on publicly available secondary data. No ethical issues were involved in the study.

## Conflicts of Interest

The authors declare no conflicts of interest.

## Permission to Reproduce Material From Other Sources

All data presented in this paper are from secondary sources that are published online.

## Data Availability

The authors have nothing to report.
